# Neuropsychological Decomposing Stroop Interference Into Different Cognitive Monitoring: An Exploratory Factor Analysis

**DOI:** 10.32598/bcn.9.10.265

**Published:** 2019-09-01

**Authors:** Mazaher Rezaei

**Affiliations:** 1. Departments of Clinical Psychology, Shahid Beheshti Hospital, Zanjan University of Medical Sciences, Zanjan, Iran.

**Keywords:** Stroop, Stroop interference, Semantic interference, Response interference

## Abstract

**Introduction:**

There are two alternative explanations of the Stroop phenomenon. Several studies have revealed that the difference in performance on congruent and incongruent trials can arise from response interference. On the contrary, many authors have claimed that Stroop interference might occur at earlier processing stages related to semantic or conceptual encoding. The present study aims to determine the number and nature of the factors necessary to account for the multiple components of Stroop interference.

**Methods:**

The sample consisted of 247 undergraduate and postgraduate students. We employed the computerized version of the Stroop task adapted to the Iranian population. An exploratory principal components analysis was conducted on the correlations of 6 variables (reaction time under congruent and incongruent conditions, omission error under congruent and incongruent conditions, and commission error under congruent and incongruent conditions).

**Results:**

Two factors were extracted. The first factor may be semantic interference, and the second factor may be response interference.

**Conclusion:**

The findings of this research are consistent with the multiple-stage account, claiming that Stroop interference is because of both semantic and response interferences.

## Highlights

- The difference in performance between congruent and incongruent conditions is known as Stroop interference.
- Stroop interference might occur at earlier processing stages because of both semantic and response interferences.
- Findings of this study are consistent with the multiple-stage account approach.

## Plain Language Summary

Various cognitive tasks measure interference between stimulus dimensions such as temporal and spatial dimensions. The most widely used task is the Stroop task. The task was originally developed by Stroop. In this task, subjects see words that denote colors (red, green, blue, and yellow) printed in a corresponding color (e.g. the word red is written in red ink) or in a non-corresponding color (e.g. the word blue is written in red ink) and they are asked to name the ink color while inhibiting the meaning of the word. When the color and the word are congruent, the task is easy; when the color and the word are incongruent, people experience interference. The Stroop interference indicates that Reaction Time (RT) is consistently longer in the incongruent conditions compared to the congruent conditions. In recent years, questions have been raised about the nature of Stroop interference. Several researchers argued that the difference in performance on congruent and incongruent trials could arise from response interference. Studies have claimed that Stroop interference might occur at earlier processing stages related to semantic or conceptual encoding. The present study aims to determine the number and nature of the factors necessary to account for the multiple components of Stroop interference. We used computerized version of the Stroop task adapted to the Iranian population. Our results confirmed that Stroop interference is because of both semantic and response interferences which is consistent with the multiple-stage account approach.

## 1. Introduction

What is required in a conflictual situation such as overcoming a habitual action in favor of an unusual one? It takes mind to involve at least 3 cognitive operations; to detect interference between 2 parallel processes, to make a decision (decide to focus on the information related to the goal and at the same time to inhibit the unrelated information), and to inhibit habitual action (to form an output code based on the appropriate decision). These complex mental operations refer to “interference” resolution. Posner et al. (Petersen & Posner, 2012; Posner, 2008, 2012a, 2012b; Posner & Dehaene, 1994; Posner & Petersen, 1990; Posner, Rothbart, Sheese, & Tang, 2007) ascribe such an operation to executive network of attention (see Petersen & Posner, 2012 for reviewing this and two other attentional networks). The executive attention network detects error and resolves interference among contradictory parallel responses (Botvinick, Braver, Barch, Carter, & Cohen, 2001; Petersen & Posner, 2012).

Various cognitive tasks measure interference between stimulus dimensions such as temporal dimensions (Nazari, Mirloo, Rezaei, & Soltanlou, 2018; Nazari et al., 2013) and spatial features (Simon & Berbaum, 1990; Simon, Sly, & Vilapakkam, 1981). The most widely studied is the classic Stroop interference between a word name and its ink color (MacLeod, 1991). The task was originally developed by Stroop. In Stroop task, the subjects see words that denote colors (red, green, blue, and yellow) printed in a corresponding color (e.g. the word red is written in red ink) or in a non-corresponding color (e.g. the word blue is written in red ink) and the subjects are required by instruction to name the ink color while inhibiting the meaning of the word. When the color and the word are congruent, the task is easy; when the color and the word are incongruent, people experience interference (MacLeod, 1991; Stroop, 1935).

The Stroop interference indicates that Reaction Time (RT) is consistently longer in the incongruent trials compared with the congruent ones (see MacLeod, 1991, for a review). The interference occurs among the automatic process of word reading and the effortful process of color naming (Augustinova & Ferrand, 2014; Cohen, Dunbar, & McClelland, 1990; Luo, 1999; Schneider & Chein, 2003). Thus, the so-called Stroop-interference denotes the ability to inhibit a usual response (i.e. an overlearned response) in favor of an unusual one (Homack & Riccio, 2004).

The variety of Stroop test versions is one of the challenges of the literature. Many later studies (Chen, Wong, Chen, & Au, 2000; Davidson & Wright, 2002; Farhadian, Akbarfahimi, Hassani Abharian, Hosseini, & Shokri, 2017; Hekmat, Alam Mehrjerdi, Moradi, Ekhtiari, & Bakhshi, 2011; Hinkin, Castellon, Hardy, Granholm, & Siegle, 1999; Rezaei, Ashayeri, Yazdandoost, & Asgharnejad, 2003; Saremi, Shariat, Nazari, & Dolatshahi, 2017) have implemented computerized stimuli that facilitate the accurate measurement of RT (Homack & Riccio, 2004). There are two types of RT measurements; the RT for the verbal response and the RT for the motor response (pressing the button). Most studies have used the first type.

In recent years, questions have been raised about the nature of processing interference. Lupker and Katz claimed that Stroop interference could occur at 4 possible stages or processes: 1. an input process; 2. a decision process; 3. a response selection process; and 4. a response output process. Two alternative explanations of the Stroop phenomenon correspond to the stages of information processing proposed by Lupker and Katz.

Several researchers argued that the difference in performance on congruent and incongruent trials could arise from response interference (Cohen, Dunbar, & McClel-land, 1990; Duncan-Johnson & Kopell, 1981; MacLeod, 1991; Roelofs, 2003). Lupker and Katz claimed that in the response selection process, an output code must be formed based on the appropriate decision (e.g. a phonetic representation in a naming task, a motor code representation in a button-pressing task, and so on). This means that the presentation of the irrelevant word automatically activates the responses with similar meanings that facilitate response selection on congruent trials but interferes with response selection on incongruent trials (De Houwer, 2003).

Several authors (Brown & Besner, 2001; Davelaar & Besner, 1988; Green, Locker, Boyer, & Sturz, 2016; Hasshim & Parris, 2015; Klopfer, 1996; Luo, 1999; Seymour, 1977; White, Risko, & Besner, 2016; Williams, Mathews, & MacLeod, 1996) have claimed that Stroop interference might occur at earlier processing stages related to semantic or conceptual encoding. Seymour debated that Stroop interference occurred because the to-be-named color and the to-be-ignored word activated two similar semantic codes.

Many analysts (Botvinick, Braver, Barch, Carter, & Cohen, 2001; Van Veen, Cohen, Botvinick, Stenger, & Carter, 2001) have claimed that the information is processed at different levels. As Van Veen et al. argued, “Theoretically, interference might occur at any or all of these levels” (Van Veen et al., 2001). There was widespread behavioral (De Houwer, 2003; Schmidt & Cheesman, 2005; Zhang & Kornblum, 1998; Zhang, Zhang, & Kornblum, 1999) and neuropsychological (Chen, Bailey, Tiernan, & West, 2011; Chen, Lei, Ding, Li, & Chen, 2013; Liu, Banich, Jacobson, & Tanabe, 2004; Melcher & Gruber, 2009; Taylor, Kornblum, Lauber, Minoshima, & Koeppe, 1997; Van Veen & Carter, 2005; Zysset, Muller, Lohmann, & von Cramon, 2001) support to give an account that both forms of interference contribute to the Stroop interference-effect.

The present study aims to determine the number and nature of the factors necessary to account for the multiple components of Stroop interference. We conducted an exploratory factor analysis to claim that we did not make a priori assumption about the nature of interference.

## 2. Methods

### 2.1. Study Participants

Zanjan University has four faculties: agriculture, Humanities, Science, and Engineering. In each of the faculties, an announcement was issued to recruit volunteer students to participate in this study. The sample consisted of 247 undergraduate and postgraduate students (Table 1).

**Table 1.** Demographic data describing subjects

**Variables**		**No.**	**%**
Gender	Female	127	48.6
	Male	120	51.4
	Total	247	100
Age (y)	18	7	2.8
	19	19	7.7
	20	47	19.0
	21	47	19.0
	22	41	16.6
	23	32	13.0
	24	21	8.5
	25	11	4.5
	26	7	2.8
	27	4	1.6
	28	4	1.6
	29	4	1.6
	30	3	1.2
	Total	247	100

### 2.2 Measure

We employed a computerized version of the Stroop task adapted to the Iranian population. The Persian version was developed by Ravan Tajhiz Sina Company. The software used the set of 4 stimuli defined by color words written in Persian alphabet; “ghermez” (Persian word for “red”), “sabz” (Persian word for “green”), “zard” (Persian word for “yellow”), and “Ābi” (Persian word for “blue”) that were presented in red, green, yellow, and blue ink. All 48 congruent stimuli were the color words shown in the corresponding ink (e.g. the word “blue” was printed in blue ink), while 48 incongruent stimuli were the color words presented in the different ink (e.g. the word “red” was written in blue ink). The output was as follows: 1. RT under congruent and incongruent conditions: interval between the perception of the color of word and pressing the colored button; 2. Omission error under congruent and incongruent conditions: the failure to respond to target button; and 3. Commission error under congruent and incongruent conditions: responses are given to non-targets button (e.g. the subject press the “red” button when the “blue” ink is presented).

Interference RT was calculated as the difference between the incongruent and congruent mean reaction times. The internal consistency of RTs was 0.6, 0.83, and 0.97 in 3 stages, respectively, by Ghadiri, Jazayeri, Ashayeri, and Ghazi-Tabatabaei. Internal consistency for error numbers were also 0.55, 0.78, and 0.79 in 3 stages, respectively. Several studies (Ghadiri et al., 2006; Hekmat et al., 2011; Rezaei et al., 2003; Saremi et al., 2017) have used the Iranian version of computerized Stroop task to study neuropsychological substrates of psychiatric disorders.

### 2.3. Study procedure

All 96 trials were randomly presented at the center of a 15-inch computer screen. The display time was 2s, and the inter-stimulus interval was 800 ms. The response buttons were labeled with color patches on the keyboard. The subjects were instructed to press the same color button as the word ink color.

### 2.4. Data analysis

The analysis of the Stroop interference was based on the assumption that there was an observed interference as measured by the task we used. Thus, we used a related t-test to examine whether the incongruent mean RT is significantly different from the congruent mean RT. Moreover, an exploratory principal components analysis was conducted on the correlations of 6 variables (Table 2).

**Table 2.** Correlations matrix of the 6 variables of computerized Stroop task

**Variables**	**Congruent Commission**	**Incongruent Commission**	**Congruent Omission**	**Incongruent Omission**	**Congruent Mean RT**
Congruent commission					
Incongruent commission	0.58^**^				
Congruent omission	0.27^**^	0.33^**^			
Incongruent omission	0.16*	0.30**	0.93**		
Congruent mean RT	0.14^*^	0.27^**^	0.56^**^	0.57^**^	
Incongruent mean RT	0.13^*^	0.31^**^	0.55^**^	0.56^**^	0.97^**^

* Correlation is significant at the 0.05 level (2-tailed).

** Correlation is significant at the 0.01 level (2-tailed).

The Kaiser eigenvalue (Kaiser, 1960) criterion (i.e. eigenvalues >1.0) was considered for determining the number of factors for extraction. The orthogonal rotation of the factors by the varimax method yielded the factor structure given in Table 3.

**Table 3.** Rotated Component Matrix

	**Component**	

	**1**	**2**
Congruent mean RT	0.902	
Incongruent mean RT	0.895	
Incongruent omission	0.845	
Congruent omission	0.823	
Congruent commission		0.904
Incongruent commission		0.847

## 3. Results

The incongruent mean RT (Mean±SD=959±175) and congruent mean RT (Mean±SD=902±161) were significantly different (t [246]=23, two-tailed P=0.001). Thus, there is Stroop interference. Two factors were initially extracted. The first factor accounted for 56% of the variance, and the second factor accounted for 23%. The first factor seems to be semantic interference and the second one is response interference. Communalities (i.e. percentage of indicator variance accounted for by the solution) ranged from 0.75 (congruent omission) to 0.82 (congruent commission).

## 4. Discussion

In the present study, we used a manual response (button press) format of the computerized Stroop task to explore the components of Stroop interfere. We found that RT was significantly longer in the incongruent condition compared with the congruent condition, indicating that the task elicited the Stroop interference. Furthermore, incongruent trials showed significantly higher commission and omission errors than congruent trials, demonstrating the task-induced Stroop interference. The present findings are consistent with other studies, which found the Stroop interference in manual response task (Ila & Polich, 1999; Schmidt & Cheesman, 2005; Sharma & McKenna, 1998; Sugg & McDonald, 1994). This finding corroborates the idea of speed-accuracy tradeoff (Fan, Flombaum, McCandliss, Thomas, & Posner, 2003; Fan, McCandliss, Sommer, Raz, & Posner, 2002; MacLeod, 1991; Stroop, 1935). It has been suggested that incongruent conditions result in longer RTs and higher error rates than congruent conditions and vice versa. As a consequence of observing Stroop interference, we achieved our purpose of conducting an exploratory factor analysis. The analysis yielded two factors.

Increased RT (both congruent and incongruent), together with omission error increasing (both congruent and incongruent) were loaded on factor one. It seems that the common features of this factor are compatible with semantic interference. Increased RT primarily reflects semantic processing, i.e. whether the concept represented by color word and the concept represented by color ink are incongruent. It seems reasonable to assume that these effortful trials indicate the subject’s ability to detect semantic interference. The adherents of semantic explanation believe that interference occurs at the earlier processing related to semantic encoding before response output. As we learned from Seymour, the to-be-named color dimension of the word and the to-be-ignored word trigger two similar semantic codes. In a nutshell, the primary explanation for the semantic competition is that Stroop stimuli trigger semantic representations of both color and word dimensions; thus, the semantic competition between these two dimensions is created before the response output (Green et al., 2016). Luo explained that color naming required the activation of an appropriate network in the verbal-lexical system. But, word reading requires only the activation of the relevant network in the lexical-verbal system, and, in this case, the operation of the semantic system is optional. The main cognitive challenge for the participants in our manual task and other versions is “to ignore or override” the semantic codes of the verbal-lexical system for responding. If the person fails to override, he or she will either respond long or will not select any response. Botvinick et al. called the type of cognitive monitoring to inhibit the verbal-lexical system (word reading) as response override (Botvinick et al., 2001).

The second finding was both congruent and incongruent commission error loaded on factor two that was called response interference. As we described, commission errors are made when responses are given to the non-targets button (e.g. the subject presses the “red” button when the “blue” ink is presented). According to Lupker and Katz, these errors can occur in the third (a response selection process) and fourth stages (a response output process). In the response selection process, an output code must be formed based on the appropriate decision (a motor code representation for the button-pressing task; e.g. the subject plan presses the “red” button when the “red” ink is presented, and to inhibit other button press).

Returning to the question posed at the beginning of this study, it is now possible to state that the findings of this study are consistent with the multiple-stage account (Chen et al., 2011; Chen et al., 2013; De Houwer, 2003; Liu et al., 2004; Melcher & Gruber, 2009; Schmidt & Cheesman, 2005; Taylor et al., 1997; Van Veen & Carter, 2005; Zhang & Kornblum, 1998; Zhang et al., 1999; Zysset et al., 2001) claiming that the Stroop interference is because of both semantic and response interferences. These studies used many modified versions of the Stroop color-word task, by which they could separate semantic interference from response interference (for example, see De Houwer, 2003 for such a modified version of the Stroop task). Van Veen and Carter used a modified version of the Stroop task, by which they could separate semantic from response interference through fMRI data. They identified that both semantic and response interference elicited independent activation in anterior cingulate and prefrontal and parietal brain regions. They concluded that the brain had discrete and parallel attentional processes for resolving these various conflicts. Other neuroimaging studies have also demonstrated two independent neural networks underlying semantic and response interferences in the Stroop task (Chen et al., 2011; Chen et al., 2013; Liu et al., 2004; Melcher & Gruber, 2009; Taylor et al., 1997; Van Veen & Carter, 2005; Zysset et al., 2001).

Neuroimaging studies (Chen et al., 2013; Egner & Hirsch, 2005; Liu et al., 2004; Ruff, Woodward, Laurens, & Liddle, 2001; Van Veen & Carter, 2005; Van Veen et al., 2001; Zysset et al., 2001) have shown that the activation of the dorsal anterior cingulate cortex in Stroop and Stroop-like tasks require people to override either a prepotent response or a rather strong conflicting dimension. These results propose the detection of interference for the dorsal anterior cingulate cortex as a definite executive function that occurs at response-related levels of processing.

It appears that the findings of the current study are limited by the use of a manual version of the Stroop task owing to its apparent motor component. Further exploratory and confirmatory factor analyses should be done to establish whether the vocal tasks support our findings, including two types of interference.

## Ethical Considerations

### Compliance with ethical guidelines

All ethical principles were considered in this article. The participants were informed about the purpose of the research and its implementation stages; they were also assured about the confidentiality of their information; Moreover, They were allowed to leave the study whenever they wish, and if desired, the results of the research would be available to them.
